# A cross-sectional study of the differences in diabetes knowledge, attitudes, perceptions and self-care practices as related to assessment of chronic illness care among people with diabetes consulting in a family physician-led hospital-based first line health service and local government health unit-based health centers in the Philippines

**DOI:** 10.1186/s12930-014-0014-z

**Published:** 2014-12-16

**Authors:** Grace Marie V Ku, Guy Kegels

**Affiliations:** Department of Public Health, Institute of Tropical Medicine, Antwerp, Belgium

**Keywords:** Biopsychosocial approach, Chronic conditions, Collaborative care, Culturally-competent care, Diabetes mellitus type 2, Family medicine principles, Perceived self-efficacy, Self-care development

## Abstract

**Background:**

The purpose of this study was to investigate differences in diabetes knowledge, attitudes and perceptions (KAP), self-care practices as related to assessment of chronic illness care among people with diabetes consulting in a family physician-led tertiary hospital-based out-patient clinic versus local government health unit-based health centers in the Philippines.

**Methods:**

People with diabetes consulting in the said primary care services were interviewed making use of questionnaires adapted from previously tested and validated KAP questionnaires and the patients’ assessment of chronic illness care (PACIC) questionnaire. Adherence to medications, diabetes diet, and exercise and the number of diabetes consultations were asked. Analysis of variance was used to determine differences in KAP, self-care practices, and PACIC and regression analysis was used to determine any associations of the abovementioned variables to the PACIC ratings.

**Results:**

A total of 549 respondents were included in the study. Differences in knowledge, attitudes, perceptions, PACIC, utilization of health services, and adherence to medications and exercise were all statistically significant. Ratings for diabetes knowledge, positive attitudes, and the perceptions of support attitudes and the abilities to perform self care, and the proportions of those properly utilizing health services and adhering to medications and exercise were higher while ratings for negative attitudes, perceived support needs, perceived support received and PACIC were lower among those consulting in the family physician-led health service.

**Conclusions:**

Combining family medicine-based approaches with culturally competent diabetes care may improve knowledge, attitudes, perceptions and self-care practices of and collaborative care with people with diabetes.

## Introduction

People with chronic conditions encounter many day-to-day situations where they have to make decisions on their own [1]; self-care plays an important role and collaboration rather than a health provider-directed care may be a more effective care model [2]. Collaborative care between people with chronic conditions and their health care providers is better achieved if the people having these chronic conditions are informed and activated [3]. Such may involve self-management education and skills development. However, the provision of self-management education and support is not simple. It does not only involve the development of self-management skills but barriers should also be addressed [4]. These barriers include personal, social and environmental barriers. Personal barriers include disease-related beliefs, emotions, knowledge and experiences [5]; socio-cultural barriers take account of the differences in language, and in cultural and ethnic beliefs and perceptions of health and illness [6] between the providers and the recipients of self-management development; and environmental barriers refer to the immediate environment of family and friends and the wider environment of the health care system and the community in supporting adoption of proper self-care behavior. Studies have demonstrated that culturally competent self-management education improved diabetes care, self-awareness and understanding of diabetes [7] while patient-centered, biopsychosocial approaches as practiced in the family medicine paradigm address personal barriers [8] and improved diabetes knowledge, patient perceived self-efficacy and glycemia [9-11].

It should be noted that self-management skills development are usually given as part of the clinical care delivered by care providers; and the background, methods, settings, and context by which self-management education and support is to be introduced and delivered should be optimized to ensure maximum absorption and adoption of self-care behavior. In settings where such self-care development activities are non-existent, establishing current status of diabetes care and its effects on factors that may affect self-care is needed to prepare these health services in the delivery of self-management education and support.

In an earlier publication, the investigators constructed a framework for self-management education and support wherein perceived self-efficacy of the person with chronic condition plays a vital role in the adoption and adherence of proper self-care behavior; this perception of self-efficacy may be affected by internal and external influences [12]. In here, the investigators theorize that healthcare providers may positively influence people with chronic conditions to perceive a higher degree of self-efficacy and to carry out self-care practices better if education and support are provided by culturally competent care providers who engage these people in a collaborative manner using the biopsychosocial approach and family medicine-based principles.

This study compared the differences in on-going primary diabetes care through assessments of chronic care delivery between a family physician-led hospital-based health service where a biopsychosocial, collaborative approach is practiced, and local government health units (LGHU) with community-based services where the care providers and recipients of care have congruent socio-cultural backgrounds, as related to diabetes knowledge, attitudes, perceptions on family support, perceptions of self-efficacy and self-care practices of people with diabetes utilizing these said services.

## Background

### Family medicine in the Philippines

Leopando and Olazo extensively discuss family medicine as a specialty in the Philippines [13]. A physician specializing in family medicine is seen as having the following roles and responsibilities: health care provider, counselor, administrator, teacher, social mobilizer and researcher. As a health care provider, the family physician assumes the roles of gatekeeper, primary care giver, hospice care giver and family health care giver. As a counselor, the family physician is expected to practice active listening skills [14] and the CEA (Catharsis, Education, Action) methods [15]. As administrator, the family physician takes on the roles of a coordinator and manager who integrates and coordinates health services for the patients and their families. As a teacher/educator, the family physician assumes the roles of trainer and health promoter providing education and skills development in family medicine and public health. As a social mobilizer, the family physician is a health advocate and a community health organizer promoting wellness and health maintenance to individuals, families and the public, and empowers patients towards self-care. As a researcher, the family physician produces relevant and evidence-based research outputs. Some family medicine practices are hospital-based while others are in health centers in the communities. In both settings, family medicine-based first line and ambulatory care services can be delivered.

Family medicine training in the Philippines is thus geared towards producing family physicians who can fulfill these roles and responsibilities. Central to this would be training not only in the provision of biomedical care but also taking into consideration various psychosocial factors that may positively or negatively affect the course of illness and how the person will participate in self-care. Training healthcare providers who are responsible for primary care on the biopsychosocial approach has long been advocated [16,17].

## Methods

This was a cross-sectional study conducted from October 2010 to September 2011 involving people with diabetes consulting at the purposively selected sites VMMC and LGHU of Batac City and Pagudpud. The main outcomes of interest were: diabetes knowledge, attitudes, perceptions, self-care practices and patients’ assessment of chronic illness care (PACIC) of the people with diabetes in the two study settings.

### The study sites

#### Family physician led-health service: the veterans health system

The *Veterans Memorial Medical Center (VMMC),* located in Quezon City, Metro Manila, is the only health service for Filipino veterans and their dependents in the whole country, to whom it delivers its free services. It is a 766-bed multispecialty hospital where all levels of services are confined in a single facility. First line health services are offered at the Department of Family Medicine and Out-Patient Services where primary care is delivered by family physicians organized as in a group practice; different clinical specialty services may also be availed of. Facilities that may deliver diabetes self-management education and support (DSME/S) are available, but there are no formal DSME/S activities.

#### Local government health units (LGHU): public health system

Public health care in the Philippines was devolved in 1992 and the responsibility of providing basic health care services for the people was handed down to local governments, specifically municipalities and cities, through their respective local government health units (LGHU) [18]. A decade before this health care devolution, the country implemented a primary health care policy which created a large cadre of community-based health workers locally called “barangay health workers” (BHW) [19]. Organizationally, the BHW fall under the governance of the barangay (village) and are selected to work in their respective areas of residence; functionally, they are under the local government health units. A BHW is assigned approximately 10–20 families and is responsible for dissemination of health information and health promotion activities, and conducts other health-related undertakings to any member of the families being attended to. The barangay is the smallest unit of government; a city or a municipality would be composed of a number of barangays. At present, a typical LGHU would be composed of at least 1 health center and a number of barangay health stations, and would have at least one physician, usually a general practitioner/non-specialist, serving as municipal/city health officer, at least one nurse and several midwives, and the cadre of BHW.

*Batac* (population 53,542 as of 2010 [20]) is a non-highly urbanized component city in the island of Luzon approximately 470 km north of Metro Manila and accessible from there by air and land transportation. The LGHU has 2 health centers. Other health care services include a tertiary-level Department of Health-operated hospital, a primary-level private hospital, a number of private multi-specialty clinics and clinical laboratories, and several private drugstores/pharmacies.

Pagudpud (population 21,877 as of 2010 [20]), the northernmost settlement in Luzon, is a rural municipality classified to be very low in economic development. It is approximately 100 km further from Batac City. It only has a basic government health unit health care. There are no laboratory facilities, nor any private clinics or drugstores/pharmacies.

All healthcare personnel of these LGHU come from the same locality where they serve; the BHW come from the same village as the families they take care of.

For the people of Batac and Pagudpud, most healthcare expenditures are out-of-pocket and formal DSME/S activities are non-existent in both the Batac City and Pagudpud government health units.

### Study participants

People with type 2 diabetes aged 20 years or more, consulting at the out-patient clinic of the VMMC or at the LGHU were invited for interview. Written informed consent was obtained from the respondents. Trained researchers conducted one-on-one interviews making use of a structured questionnaire testing diabetes knowledge and inquiring on attitudes, perceptions, PACIC, health-seeking behavior and health care practices.

### Diabetes knowledge

A 24-item questionnaire on diabetes knowledge, answerable by yes, no, or I do not know, was prepared based on the Fitzgerald et al. Brief Diabetes Knowledge Test [21] and the Garcia et al. Diabetes Knowledge Questionnaire [22], adjusted to the local context with regard to locally available medications and/or remedies, local food, and the local epidemiology of diabetes and its complications, taking into consideration local norms, customs, values, and traditions. Diabetes knowledge was measured as the proportion of correct answers to the knowledge questionnaire.

### Attitude and perceptions

Questions on attitudes and perceptions were adapted from the survey questionnaires of the University of Michigan Diabetes Research and Training Center [23,24]. The questions were formulated as statements to which answers made use of a 5-point Likert scale ranging from a lowest rating of 1 (strongly disagree/never) to a highest rating of 5 (strongly agree/always). Negative and positive attitudes were measured separately. We inquired into perceived needs for self-care support, support received, support attitudes as well as perceptions related to the person’s ability to perform self-care, namely, to control blood glucose, control weight, do things needed for diabetes (diet, exercise, taking medications), and handle feelings about diabetes. Questions on support needed and received were directed towards support a person with diabetes needs and receives from family and friends. Questions on support attitudes were about the perceptions of how a person with diabetes is being treated, accepted and supported by family and friends. The perceived ability to perform self-care was equated to feelings of self-efficacy. Positive attitudes, negative attitudes, perceived support needs, etc. were defined as a rating of more than 3. Perceived support received was said to be congruent to perceived support needs if the difference between the former and the latter was 0; congruence <0 means that the perceived support received was less than the perceived needs while congruence >0 means that support received was more than the perceived needs.

### Assessment of chronic illness care

Glasgow’s Patient’s Assessment of Chronic Illness Care (PACIC) was used [25]. The PACIC is a 20-item questionnaire making use of a 5-point Likert scale with 1 (almost never) as the lowest and 5 (almost always) as the highest rating and where the questions can be grouped together to form the subscales “patient activation”, “delivery system design”, “goal setting”, “problem solving”, and “follow-up/coordination”, which are linked to Wagner’s chronic care model elements [3] and are related to the provision of collaborative care. Good ratings were defined as a rating of more than 3.

### Self-care practices

In this study, the questions on self-care practices referred to health seeking behavior in terms of frequency of consultations done for diabetes, and adherence to medications, diet and exercise. The question on consultation was about the number of times the person consulted for diabetes with any formal health care provider in the past 6 months, and was stratified as none or once for the past 6 months (0-1/6 months) and 2 or more for the past 6 months (≥2/6 months). The latter was interpreted as the more adequate practice in this setting. Questions on medication adherence were about the medications as prescribed by care providers and if the respondents were taking the right medications at the right dosages at the right times; these were answerable by “no” or “yes” and summarized as “no” if any of the questions were answered with “no” and “yes” if all the questions were answered with “yes”. The question on diet adherence was answerable by “no”, “sometimes”, or “yes/always”; “no” and “sometimes” answers were later combined and transformed to “no/not fully adherent”. Questions on exercise asked on the type, frequency, and duration of exercise done; the answers were then transformed to “no” or “yes” based on the criteria of doing 150 minutes of moderate-intensity aerobic physical activity or at least 75 minutes of vigorous-intensity aerobic physical activity throughout the week [26].

### Statistical analysis

Statistical analyses were done making use of the statistical package Stata/IC version 11.0 [27].

Initial statistical analysis showed significant variations in age and gender between the two groups and the potential of these factors as confounders; thus, data collected were adjusted for age and gender based on the 2010 Philippine population [20] and Stata survey statistics were applied. Differences in diabetes knowledge, positive and negative attitudes, fear of diabetes, perceived support needs, perceived support received, support attitudes, the perceptions of the abilities to perform self-care and the PACIC and its subscales according to the type of health service utilized were tested using the regress command [28]. The two-way tabulate command, which makes use of Pearson’s chi-square, was applied on the adjusted data to determine significant differences between the health services based on proper utilization of health services and adherence to medications, diet and exercise [29].

Logistic regression analysis was used to determine any associations of the ratings of the PACIC and its subscales and knowledge, attitudes and perceptions rating; adherence to medications, diet and exercise; and utilization of health services. Level of education, known duration of the condition and the study settings were considered as additional potential confounders. Bivariate analysis was initially done using a significance cut-off of 5%. Variables fulfilling the criterion were then analyzed in multivariate logistic regression, with step-wise exclusion of variables having an alpha >0.05 to arrive at the final models.

Cronbach’s alpha was used to measure the internal consistency/validity of the questions asked.

## Results

**Figure 1:**
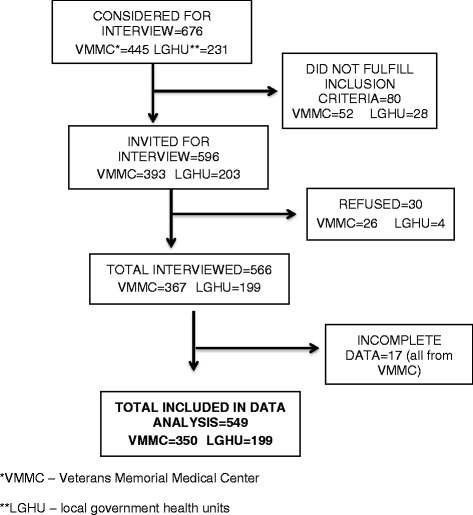
**Flow of recruitment and inclusion for interview and analysis.**

A total of 549 respondents were interviewed: 350 from VMMC and 199 from the LGHU. Figure 1 shows the flow of inclusion of the study participants and Table 1 lists some demographic characteristics of the respondents.

**Table 1:** **Demographics of the respondents**

		**All**	**VMMC**	**LGHU**
Number of respondents, n (%)		549	350 (63.8%)	199 (36.2%)
Gender (Male), n (%)		227 (41.4%)	176 (50.3%)	51 (25.6%)
Age in years, mean (range)		62.8 (27 – 92)	65.7 (33 – 92)	57.6 (27 – 90)
Duration of diabetes in years, mean (range)		7.0 (0.5 – 37)	8.3 (0.5 – 37)	4.7 (0.5 – 35)
Education, n (%)	0-6 years	126 (23.0%)	69 (19.7%)	57 (28.6%)
	7-10 years	219 (39.9%)	145 (41.4%)	74 (37.2%)
	>10 years	204 (37.1%)	136 (38.9%)	68 (34.2%)

Internal consistency/reliability of the knowledge, attitudes and perceptions (KAP) questions in this study population ranged from 0.72 to 0.94 [12]; it was 0.93 for the PACIC questions.

### Differences in KAP, PACIC and self-care practices

Table 2 lists the adjusted means and regress command p values of the different variables comparing the two study settings. The differences in knowledge, attitudes, perceptions and self-care practices among people with diabetes consulting in the VMMC and the two LGHUs have been presented and discussed in a previous publication [12]. Among those consulting at the family physician-led hospital health service, the age- and gender-adjusted ratings on the knowledge test, positive attitudes, perceived support attitudes and the perceived abilities to perform self care/perceptions of self-efficacy and the proportions of those properly utilizing health services and adherent to medications and exercise were significantly higher and the ratings for negative attitudes were significantly lower. Although the rating for perceived support received was lower among those consulting at the family physician-led health service, the rating for perceived support needs was also lower; and congruence of the support needs to the support received, although statistically insignificant, was better.

**Table 2:** **Age- and gender-adjusted mean (95% confidence intervals) KAP and PACIC, and p values of the differences in KAP and PACIC of people with diabetes consulting at the family physician-led hospital-based (VMMC) vs. local government-based (LGHU) first line health services**

**Factor**		**VMMC**	**LGHU**	**P value (using regress command)**
Diabetes knowledge		70.7 (63.5-77.9)	58.7 (54.9-62.9)	<0.001
Positive attitudes		3.7 (3.5-3.8)	3.3 (3.2-3.4)	0.002
Negative attitudes		2.2 (1.9-2.6)	3.1 (2.8-3.5)	0.001
Fear		2.6 (1.9-3.4)	3.5 (2.8-3.5)	0.076
Perceived support needs		2.7 (2.2-3.1)	4.3 (3.9-4.7)	<0.001
Perceived support received		3.5 (3.1-4.0)	4.4 (4.2-4.6)	<0.001
Congruence of perceived support received to perceived support needs		0.89 (0.18-1.61)	0.11 (−0.38-0.60)	0.095
Perceived support attitudes		5.0 (5.0-5.0)	4.6 (4.4-4.8)	<0.001
Perceived ability to perform self-care/Perceptions of self-effciacy	Overall	3.7 (3.5-3.9)	3.2 (3.0-3.4)	<0.001
	To control blood glucose	4.3 (4.1-4.5)	3.2 (2.8-3.6)	<0.001
	To control weight	4.3 (4.1-4.5)	3.5 (3.2-3.8)	<0.001
	To do things needed to be done for diabetes	4.1 (3.8-4.3)	3.3 (3.0-3.6)	<0.001
	To handle feelings on diabetes	4.0 (3.8-4.3)	3.5 (3.2-3.8)	0.030
Patients’ assessment of chronic illness care	Summary of overall score	2.6 (2.1-3.2)	3.2 (3.1-3.3)	0.016
	Patient activation	2.6 (1.6-3.6)	3.5 (3.4-3.7)	0.086
	Delivery system design	3.3 (2.5-4.0)	3.6 (3.4-3.8)	0.465
	Goal setting	2.6 (2.0-3.2)	3.1 (3.0-3.3)	0.064
	Problem solving	3.0 (2.7-3.2)	3.3 (3.1-3.5)	0.042
	Follow-up/coordination	2.1 (1.8-2.3)	3.0 (2.9-3.2)	<0.001

Age- and gender-adjusted total PACIC ratings were higher in the LGHU-based primary care services. Analysis of the subscales of the PACIC shows that the ratings for “problem solving” and “follow-up and coordination” were significantly higher among those consulting at the LGHU.

The proportions of people with proper utilization of health services and adherence to medications and to exercise were bigger among those consulting at the VMMC (Table 3).

**Table 3:** **Proportion of people with diabetes consulting at the health services with good self-care practices, adjusted to age and gender**

**Self care practice**	**VMMC**	**LGHU**	**P value (Pearson’s chi** ^**2**^ **)**
Utilization of health services	78.8%	37.0%	<0.001
Adherence to medications	93.7%	52.4%	<0.001
Adherence to diet	61.6%	47.9%	0.924
Adherence to exercise	66.1%	32.2%	<0.001

### Associations between KAP, PACIC, perceived self-efficacy & self-care practices

Analysis of possible associations of self-care practices with the PACIC and subscales ratings showed a significant positive association between the PACIC summary score rating and medication adherence (OR = 1.5185, p = 0.030); and a positive association between the PACIC subscale “delivery system design” and diet adherence (OR = 1.3650, p = 0.022). In the earlier study [12], perceived self-efficacy was identified to be associated with all four self-care practices with the final models’ odds ratios and p values of perceived self-efficacy as follows: utilization of health services OR = 1.784, p = 0.002; medication adherence OR = 1.611, p = 0.012; diet adherence OR = 2.015, p < 0.001; and exercise adherence OR = 1.635, p = 0.001.

Logistic regression analysis of perceived self-efficacy showed positive association with the PACIC summary (OR = 1.8798; p < 0.001) and the subscale “patient activation” ratings (OR = 1.777; p < 0.001); while positive associations with the setting VMMC (OR = 3.823, p < 0.001), diabetes knowledge (OR = 4.258; p = 0.025) and positive attitudes (OR = 1.747;p = 0.001) were identified in the earlier study [12].

## Discussion

There were no existing formal diabetes self-management education and support activities in both family physician-led and local government-based health services at the time of this cross-sectional study. However, in general, family physicians practice active listening and counseling skills and patient-centered biopsychosocial approaches; other health care personnel likewise conduct one-on-one and group health teachings to people consulting at the VMMC. At the LGHU, physicians or nurses generally conduct clinical consultations at the health centers while BHW, and occasionally the midwives, follow-up through home visits of the families under their care.

This and the previous study conducted [12] have demonstrated that increased diabetes knowledge, positive attitudes, the study setting VMMC, and higher PACIC summary and the PACIC subscale “patient activation” ratings are associated with higher levels of perceived self-efficacy. Perceived self-efficacy is positively associated with all four self-care practices. Additionally, a higher PACIC summary rating is associated with adherence to medications while a higher rating for the PACIC subscale “delivery system design” is associated with diet adherence. People consulting at the study setting LGHU gave higher ratings for the PACIC and its subscales. With these, the specific characteristics of the two study settings were analyzed further.

### Family medicine-led health service (VMMC)

It seems that the patient-centered, active listening-based, biopsychosocial approaches practiced by family physicians at the VMMC contribute to better knowledge, attitudes and perceptions among people with diabetes [30,31] as was corroborated by this research. Diabetes self-care knowledge and skills development may be an unmet need in non-patient-centered care settings [32]. Adopting self-care entails changes in behavior. Such behavior change should be understood as part of an interpersonal process that may be enhanced by a collaborative, patient-centered approach and an effective and clear communication process between the health care provider and the person with chronic condition [2]. This was demonstrated by a significantly higher perception of self-efficacy and better self-care practices in terms of utilization of health services, adherence to medications and adherence to exercise regimen among people consulting at the family physician-led health service. Furthermore, the active listening and CEA approaches actually employed during clinical consultations and in patient and family counseling sessions may be useful in exploring and addressing the reasons behind why, how, and when people with chronic conditions do not engage in adequate self-care. This may help eliminate environmental barriers such as conflicted family relationships, which have been shown to adversely affect self-care [33]. The better perception of support attitudes of family and friends among those consulting at the family physician-led health service may connote to better resolution of identified family dysfunctions and pathologies as practiced in family medicine. Furthermore, the higher degree of support being offered at the VMMC in terms of free medications and laboratory tests and the availability of specialty services would favor better self-care practices, especially medication adherence as was seen in this study. Interestingly, a higher PACIC summary rating was likewise associated with medication adherence.

### LGHU with culturally-competent healthcare workers

PACIC ratings were higher among those consulting at the LGHU. It may be that respondents from the LGHU-based health services do not have high expectations or may not be knowledgeable enough to have such expectations form their health service, thus their higher PACIC ratings. More than that, the socio-cultural homogeneity of the health care workers serving people with diabetes within their own communities in the LGHU-based health service may have played a role especially because only the subscales “problem solving” and “follow-up and coordination” were noted to have significantly higher ratings among those consulting at the LGHU health services. Socio-cultural barriers exist in the family physician-led health service as patients come from all over the country and, as is typical in group practices, may be seen by a different health care worker each time they consult. “Problem solving” may be viewed to be better if the person with diabetes collaborates with a health care worker without any socio-cultural barriers [34], which is the case in the LGHUs. The perception of better “follow-up and coordination” may be enhanced by the home visits done by the BHW.

### Combining family medicine principles and cultural competence

Although people consulting at the VMMC may have higher knowledge and perceived self-efficacy ratings, and perceived self-efficacy is positively associated with the four self-care practices, two self-care practices were likewise positively associated with the PACIC score; these findings imply that certain qualities in both the Veterans health system and the LGHU may contribute to the adoption and adherence to self-care.

**Figure 2:**
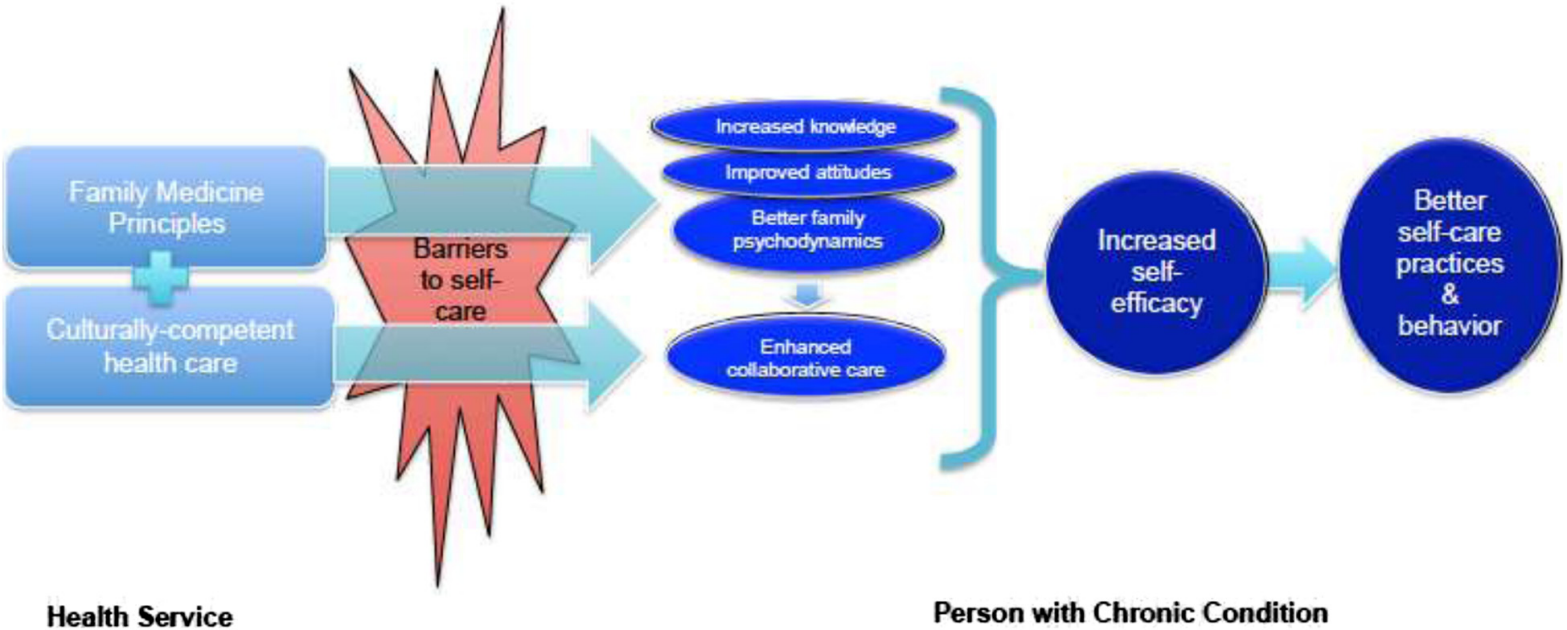
**Theoretical framework: using culturally-competent, family medicine principles-basedchronic care delivery for self-care development.**

Ideally, self-management education and skills development should be carried out by a team of professionals, which include primary care physicians, specialists, nurses, nutritionist/dietitians, psychologists [35]. However, such professional composition and creation of teams concentrating solely on chronic care delivery are not possible across all areas in low- and middle-income countries (LMIC). Although LMICs may have tertiary medical services or referral centers where the abovementioned resources are available, such health services, such as the VMMC in the Philippines, are mainly concentrated in urban areas. In a wider portion of these countries, professional health care providers are limited to non-specialist physicians or nurses. Considering this context, a collaborative biopsychosocial approach practiced by a care provider having a similar socio-cultural background would be preferred to engage the person with chronic conditions to adopt and adhere to self-care behavior. Such an approach is consistent with family medicine principles and is compatible with the current organization of LGHUs in the Philippines. However, in most cases, LGHU personnel, from the physicians to the lay health care workers, do not possess the skills and training relevant to family medicine practices. This may be addressed by training LGHU staff on family medicine-based care such as patient-centered, biopsychosocial approaches and the use of the CEA methods and active listening skills. Figure 2 proposes a theoretical framework on how combining culturally competent health care with family medicine approaches may increase self-efficacy and improve self-care behavior.

### Conclusions and recommendations

This study has demonstrated that homogeneity in the socio-cultural backgrounds of people with diabetes and their health care providers seems to play an important role in the assessment of chronic illness care delivery while practicing a patient-centered, biopsychosocial approach, and active listening and counseling skills seems to result in better diabetes knowledge, improved attitudes, and better handling of familial psychodynamics leading to better perceptions of self-efficacy and self-care practices.

Culturally appropriate diabetes care that extends from the clinics to the community provided by health care personnel with similar socio-cultural backgrounds may bring about better collaborative care with people with diabetes. On the other hand, family medicine-based approaches may contribute to improved knowledge, attitudes, perceptions, and self-care practices of people with diabetes. Mutual enhancement of both family medicine professionalism and cultural competence is instrumental in promoting self-efficacy and adoption of adequate self-care behavior in people with diabetes.

## Abbreviations

BHW: Barangay (village) health worker
CEA: Catharsis, education, action
DSME/S: Diabetes self-management education and support
KAP: Knowledge, attitudes and perceptions
LGHU: Local government health unit
PACIC: Patients’ Assessment Of Chronic Illness Care
VMMC: Veterans Memorial Medical Center
